# Characteristics of Commercial and Raw Pellets Available on the Italian Market: Study of Organic and Inorganic Fraction and Related Chemometric Approach

**DOI:** 10.3390/ijerph20166559

**Published:** 2023-08-11

**Authors:** Pietro Pandolfi, Ivan Notardonato, Sergio Passarella, Maria Pia Sammartino, Giovanni Visco, Paolo Ceci, Loretta De Giorgi, Virgilio Stillittano, Domenico Monci, Pasquale Avino

**Affiliations:** 1 Department of Biomedicine and Prevention, University of Rome, Tor Vergata, 00155 Rome, Italy; ppfpcd@tin.it; 2 Department of Agricultural, Environmental and Food Sciences (DiAAA), University of Molise, 86100 Campobasso, Italy; ivan.notardonato@unimol.it (I.N.); sergio.passarella@studenti.unimol.it (S.P.); domenico.monci@unimol.it (D.M.); 3 Department of Chemistry, University of Rome “La Sapienza”, 00185 Rome, Italy; mariapia.sammartino@uniroma1.it (M.P.S.); giovanni.visco@uniroma1.it (G.V.); 4 Institute of Atmospheric Pollution Research, Division of Rome, c/o Ministry of Environment and Energy Security, 00147 Rome, Italy; ceci@iia.cnr.it (P.C.); degiorgi@iia.cnr.it (L.D.G.); 5 Istituto Zooprofilattico Sperimentale del Lazio e della Toscana “M. Aleandri”, 00178 Rome, Italy; v.stillittano-esterno@sanita.it; 6 Institute of Atmospheric Pollution Research (IIA), National Research Council (CNR), Rome Research Area-Montelibretti, 00015 Monterotondo, Italy

**Keywords:** pellet, wood, biomass, atmosphere, outdoor, indoor air quality, VOCs, PAHs, heavy metals, chemometrics

## Abstract

Air pollution and the increasing production of greenhouse gases has prompted greater use of renewable energy sources; the EU has set a target that the use of green energy should be at 32 percent by 2030. With this in mind, in the last 10 years, the demand for pellets in Italy has more than doubled, making Italy the second largest consumer in Europe. The quality of the pellets burned in stoves is crucial to indoor and outdoor pollution. Among other parameters, moisture and ash are used to classify pellets according to EN ISO 17225:2014. This work involved the analysis of the organic and inorganic fraction of both some finished products on the Italian market and some raw materials (e.g., wood chips) sampled according to the technical standard EN 14778:2011. The analytical results showed the presence of some substances potentially harmful to human health such as formaldehyde, acetone, toluene and styrene for the organic fraction and nickel, lead and vanadium for the inorganic fraction. The chemometric approach showed that it is the inorganic fraction which is most responsible for the diversification of the samples under study. The detection of some substances may be a warning bell about the impact of such materials, both for the environment and for human health.

## 1. Introduction

The increase in atmospheric greenhouse gases such as CO_2_ (from 280 ppm to 400 ppm) [1] has prompted nations to introduce regulations to encourage the use of renewable energy. The European Union (EU) set a target to use 20 percent renewable energy by 2020 [2] and then to reach 32 percent by 2030 [3], while the World Energy Council has estimated that the use of renewable energy from biomass will triple in 2050 compared to the levels used in 2010 [4] in order to reduce greenhouse gas emissions [5,6,7]. Currently, biofuels can be classified into solids (wood, wood chips, pellets and coal) [8], liquids (bioethanol, biodiesel, pyrolysis bio-oil and drop-in fuels) and gases (biogas and syngas) [1]. Among solid biofuels, pellets were developed to reduce some critical factors of wood and wood chips such as low energy density and high moisture, which impact the efficiency of use and transportation too much [7]. The spread of pellets initially took place in the USA, Denmark, Sweden and Austria, and later in other countries to help reduce emissions. Pellets, precisely because of their ease of transport and storage, have developed especially in rural areas where a gas distribution network is often absent [4,8,9]. The popularity of pellets as a renewable energy source is due to their many environmental benefits: for example, CO_2_ produced through combustion can be considered zero since it has previously been adsorbed into the plant during its life cycle. In 2008, the EU emitted 12.6 million tons less CO_2_ by replacing part of coal and fuel oil with pellets [5,7]. The environmental benefits are accentuated if pellet production is derived from local forests, minimizing emissions from transportation [5,10]. In addition, pellets produce fewer emissions than coal and less ash that can be enhanced as fertilizer [5,11,12].

The production and consumption of pellets increased sharply between 2000 and 2017. Europe, followed by North America, is the world’s largest producer and also consumer of pellets [7]. In Europe, pellets are used for both heat and electricity production [13]. The largest consumer in the EU is Italy, which went from a pellet consumption of 1.4 Mt in 2013 to about 2.25 Mt in 2015 [7] and 3.4 Mt in 2019 [14]. However, Italy’s pellet production is not sufficient to meet the current demand, so it is forced to import from other countries, including Austria and countries in Eastern Europe [7,15].

Pellets are usually produced from biomass from wood industry production waste (sawdust and shavings) and pruning residues. The preeminent stage of pellet production is the extrusion of wood chips medially using mechanical pressure. At this stage, high temperatures (about 90 °C) are generated that allow the lignin to plasticize and bond the pellet in the subsequent cooling stage, ensuring the integrity of the pellet itself [1,7,10]. The resulting product has a diameter between 2 and 12 mm and length of about 20 mm, a moisture content between 5 and 10 percent and a density of 650 kg m^−3^ [1,7]. To improve the integrity of the pellet, between 0.5 and 1 percent adhesives such as potato starch and lignosulfonates can be added, the latter being responsible for higher sulfur emissions into the environment [11]. Their addition, and the possible presence of chemical contaminants, can generate health concerns following pellet combustion. Although classified as a biofuel, the use of pellets also generates environmental pollution concerns [11].

Pellet quality is a very important factor because it affects the quality of combustion, emissions and efficiency of the combustion process [5]. In particular, the ash content within the pellet is important for boiler operation. Slag deposits can indeed be created [13]. An important mineral is chlorine, which promotes environmental emissions of dioxins and chlorophenols that are generated during pellet combustion [11,16]. A risk that impacts indoor and environmental pollution is the release of chemicals from pellet combustion within domestic stoves that increase health risk in terms of pulmonary and cardiovascular diseases [17,18]. For this reason, both the quality and maintenance of the boiler and the quality of the pellets are extremely important [5]. The regulation of pellet standards developed at the European level with the introduction of UNI EN 14961:2011 [19] by the European Committee for Standardization (CEN), which was updated in 2014 with EN ISO 17225:2014 [20]. According to this certification, pellets can only be produced from certain raw materials such as (a) forests, plantations and other virgin wood; (b) by-products and residues from the wood processing industry; (c) used wood that has not been chemically treated; (d) herbaceous biomass; (e) fruit biomass and (f) aquatic biomass. Italy has also adopted such guidelines (Pellet Gold quality certification), with the determination of formaldehyde and radioactive isotope content also guaranteeing a pellet free of additives and chemical contaminants [7].

This work aims to identify and quantify the presence of exogenous, organic and inorganic chemical substances in commercial pellets and raw materials on the Italian market, so as to verify their quality by highlighting their impact on human and environmental health. To the authors’ knowledge, this is one of the first works that moves in this direction.

## 2. Materials and Methods

### 2.1. Sampling

The samples analyzed are as follows: olive pomace (bulk) #1, olive pomace (bulk) #2, wood sawdust #3, wood sawdust #4, wood chips (pruning and wood waste) #5, logs (wood briquettes) #A, briquettes (wood briquettes) #B, briquettes (wood briquettes) #C, briquettes (wood briquettes) #D, Canadian pellets #E, Romanian pellets #F, pellets #G, pomace peanuts #H, briquettes (pomace briquettes) #I, Romanian pellet #L, Romanian pellet #M, pellet #N, pellet #O. Samples indexed with numbers were sampled according to the UNI EN ISO 18135:2018 [21], whereas samples indexed with letters (13 samples from sample letter A to sample letter O) were taken in original unopened packages.

### 2.2. Analytical Methods

All metals and all compounds were analyzed according to the official methods reported in Table S1 of the Supplementary Materials.

Specifically, we analyzed the following:

- 9 aldehydes (namely formaldehyde, acetaldehyde, acrolein, crotonaldehyde, *iso*-valeraldehyde, valeraldehyde, propionaldehyde, butyraldehyde and *iso*-butyraldehyde, hexanal): the method provides procedures for the determination of free carbonyl compounds in various matrices through derivatization with 2,4-dinitrophenylhydrazine (DNPH) [22];
- physical state (pH, residue at 105 °C, residue at 600 °C, total organic carbon TOC, sulfides) [23];
- total cyanides: this method is designed for the extraction of soluble and insoluble cyanides [24];
- 30 metals (i.e., Al, Ag, As, B, Be, Ca, Cd, Co, total Cr, Cr VI, Cu, Fe, Hg, K, Mg, Mn, Mo, Na, Ni, Pb, Sb, Se, Si, Sn, Ta, Te, Ti, V, Zn): inductively coupled plasma-atomic emission spectrometry (ICP-AES) was used to determine trace elements in solution [25];
- 10 organic aromatic compounds, 23 chlorinated aliphatic compounds and 6 halogenated aliphatic compounds: among the most commonly used techniques for volatile organic analytes, purge-and-trap followed by GC-MS analysis was used [26];
- 22 PAHs, 34 organochlorinated pesticides and 20 organophosphorous pesticides: direct injection of each sample followed by GC-MS analysis was involved for analyzing such compounds [27].

Leaching tests were also carried out on the sample of “wood chips” according to the UNI standard: the sample was placed in contact with demineralized Dionex water for 24 h. Subsequently, analyses were performed for the determination of metals (arsenic, barium, cadmium, total chromium, copper, mercury, molybdenum, nickel, lead, antimony, selenium, zinc, chlorides, fluorides, sulphates) and the content of dissolved organic carbon present in the water sample.

## 3. Results

### 3.1. Preliminary Analysis: Radioactivity Analysis

The radioactivity of all the samples was evaluated before the analysis was carried out, as products from different parts of Europe were present. Radioactivity determination was carried out on both the raw materials and the pellets. In 2009, following analysis of Lithuanian imported pellets, a contamination of Cesium-137 was discovered, most likely due to the Chernobyl accident. Because there is a high consumption of pellets from abroad in Italy, even small amounts of radioactivity in kilograms of pellets can become a public health problem. To exceed the critical threshold of 10 µSv∙year^−1^, the pellet should show 1200 Bq∙kg^−1^, and such data were never found [15]. All the samples we analyzed showed no radioactivity.

### 3.2. Chemical Analysis

The analyses conducted cover a fair number of samples and investigate many parameters. The complete dataset showing the values of the analyses carried out for each sample under investigation can be found in Table S1 of the Supplementary Materials.

Table S2 of the Supplementary Materials shows descriptive statistics of all parameters. This table provides a summary description of the values obtained for each parameter as a result of the analysis of the samples. The results are explained in the following paragraphs. Here, the authors wish to underline the wide range of some parameters under study. The element presenting the greatest variation among the samples is Calcium; the other species presenting a high variation are Aluminum, Iron, Magnesium, Potassium, Sodium and the sum of C_10_–C_40_ hydrocarbons. Considering the different product categories of the samples, these data must be commented on in critical terms in the following paragraphs.

#### 3.2.1. Chemical and Physical Parameters

The analysis of the samples initially focused on some general parameters such as pH. For all samples, the value presents a range between 3.7 and 5.6, except for the wood chip sample, which presents a value of 7.8, the maximum value. The sample under consideration may represent a danger to human health because it could generate an alkaline aerosol, which could be responsible fora the development of particular phenomena of pollution, as occurred in 2020 with COVID-19, due to the alkaline pH of the aerosol itself [28]. Regarding humidity, the values have a wide range between 4.3 and 46.3 percent, while those of total mineral fraction, represented by dry matter at 600 °C, range between 0.32 and 4 percent. These two parameters are very important for the classification of biomass for biofuels [29]. The amount of ash is important for the purpose of determining the quality of pellets, because it affects both the quality of combustion in terms of calorific value (reducing it as also in the case of moisture) and in terms of environmental emissions. In addition, some chemicals of the mineral fraction provide indications of the type of raw material by differentiating pellets made of wood from those from other types of biomasses, such as waste, and within the woody fraction, such as that derived from sawdust from that of fresh-cut residues (which are more exposed to pollution in the winter season) [11,30,31,32]. In particular, both moisture and ash data for sample #5, shown in Table S1, are very high, which, because of the above, may suggest relatively recent pruning waste with a high proportion of bark such that the ash value goes beyond the UNI EN 17225 for B quality grades [29]. For the pomace hazel samples, although there is no European standard, the moisture values would be outside the quality parameters of the UNE 164003 [33].

#### 3.2.2. Inorganic Moiety

**Figure 1:**
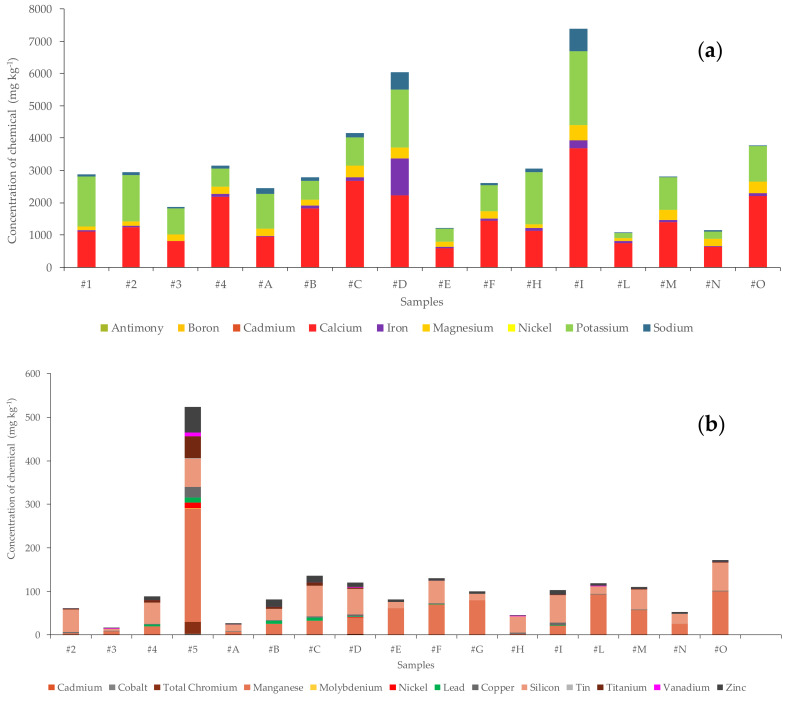
Graphics display the element concentrations present in the samples at high (**a**) and low levels (**b**).

The inorganic fraction analysis evidences that elements such as calcium, potassium, iron, magnesium, aluminum, sodium, manganese, silicon, boron, zinc and barium are common to all samples. On the other hand, elements such as mercury and chromium VI, extremely toxic/harmful species, are not present in any samples. Figure 1a shows the concentration of the most common elements present in the samples: calcium, potassium, magnesium, iron, sodium and aluminum are expressed in mg kg^−1^. Figure 1b shows the concentration of the other elements present in the samples.

**Figure 2:**
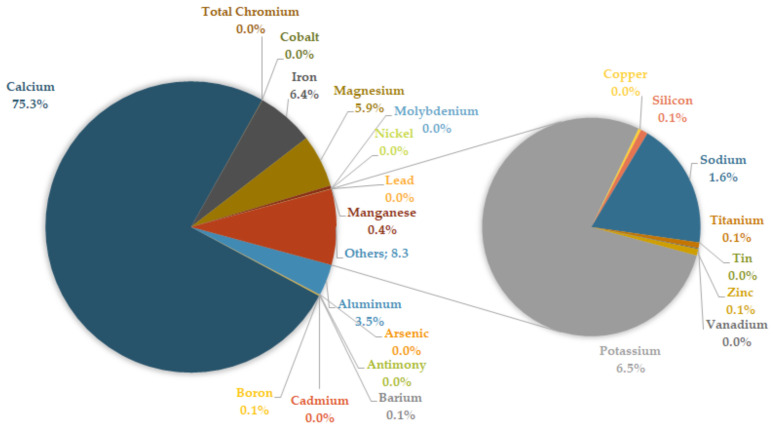
Percentage composition of inorganic fraction in sample #5.

The above graphs deliberately do not contain data for sample #5. This choice is due to the high concentration of the inorganic fraction in this sample, which would push down the bar columns of the other samples. The mineral fraction data of sample #5 (pruning waste and wood) show both higher levels and a more complete spectrum of the inorganic fraction than all other samples. Figure 2 shows the percentage of the mineral components forming sample #5.

Some toxic metals were found, such as nickel in samples in samples #D (briquette), #F (Romanian pellet) and #I (briquette); lead in samples #4 (wood sawdust), #B (briquette), #C (briquette), #D (briquette), #F (Romanian pellet), #I (briquette), #L (Romanian pellet) and #O (pellet) and vanadium in all samples except samples #E, #G and #N. All three of these compounds were also found in sample #B (wood chips, pruning waste and wood).

The presence of nickel in the environment could be due to airborne dust, rock erosion and volcanic eruptions, as far as natural phenomena are concerned, and the combustion of fuels (additives) [9] and waste [30] for anthropogenic ones. The presence of lead in the environment is due to its use in the production of boats, pipes, paints and ceramics and in battery recycling [34]. The presence of vanadium in the ambient air is from the combustion of fossil fuels used by transportation vehicles and heating plants [35].

#### 3.2.3. Organic Fraction: Volatile Organic Compounds (VOCs) and Polycyclic Aromatic Hydrocarbons (PAHs)

**Figure 3:**
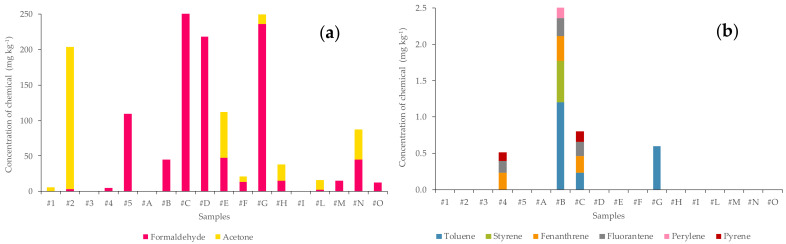
Graphics display the concentration of formaldehyde and acetone (**a**) and some volatile organic compounds (VOCs) (**b**) present in the samples.

In terms of the presence of organic compounds found in the samples examined, as can be seen from Figure 3a, the compounds most commonly present are formaldehyde and acetone. In Figure 3b, only other molecules found in the samples are shown.

Formaldehyde ranges from a minimum level of 2.48 mg kg^−1^ in sample #L (Romanian Pellets) up to values of 480 mg kg^−1^ in sample #C (briquettes), going through values of 218 mg kg^−1^ for sample #D (briquettes), 236 mg kg^−1^ for sample #G (pellets), 109 mg kg^−1^ for sample #5 (wood chips) and so to decrease. Only samples #1 (pomace pit), #3 (wood sawdust), #A (briquettes) and #I (pomace briquettes) have no formaldehyde levels. Since 1962, from Wittmann’s study of formaldehyde release from chipboard, formaldehyde has been considered as an indoor air pollutant [36]. Nowadays, it is considered a ubiquitous pollutant. The presence of formaldehyde in the indoor environment is often attributed to wood panels coated with urea resin produced by polymeric condensation of urea and formaldehyde, which, however, tends to hydrolyze under humid and acidic conditions, releasing formaldehyde into the environment [37]. The samples under consideration could have been made from non-virgin wood waste as required by Italian regulations. The second organic compound present is acetone. This was found in samples #1, #2 and #H (all pomace wood chips) and in samples #E (Canadian pellets), #F (Romanian pellets), #G (pellets), #L (Romanian pellets) and #N (pellets). Specifically, a concentration of 200 mg kg^−1^ of acetone was found in sample #2, which could be due to the treatment of olive pomace with aqueous solutions and solvents such as acetone to separate the kernel from the olive pulp [38]. For pellet samples, on the other hand, the presence of acetone could result from its use as a solvent in both paints and paint strippers, leading to a raw material unsuitable for pellet production [39,40].

Also of interest is the presence of toluene in samples # B (briquettes), #C (briquettes) and # G (pellets), although it was detected in low amounts (0.23 to 1.2 mg kg^−1^). One of the most important operations in wood concerns its consolidation. Consolidants are substances designed to re-establish, generally by impregnation, a sufficient degree of cohesion in materials that, due to decay, have gradually come to lose that condition of aggregation that originally characterized them. Consolidants can be natural, such as beeswax, [41,42] and synthetic, such as paraloid B72, formed from a 5% solution in toluene/isopropanol. Impregnations are generally performed using the “wet” technique until completely saturated [43]. The contamination of wood by toluene can be explained by the use of previously consolidated wood.

On the other hand, styrene was found in only one sample, #B (briquettes). At room temperature, it is an oily transparent liquid with a characteristic sweetish odor. It is toxic, flammable and insoluble in water and dissolves in most common organic solvents. [44]. Styrene used for the production of polymers generates health hazards. Its metabolite, styrene-7,8-oxide (SO), results in the formation of protein adducts in RNA and DNA that alters the ability of DNA to repair. Oxidative stress is created, leading to genotoxic risk [45]. For this reason, styrene was officially recognized as a carcinogen by the Twelfth Report on Carcinogens, published on 10 June 2011 by the U.S. Dept. of Health, National Toxicology Program. The styrene molecule is used in the production of polymers and copolymers such as polystyrene and acrylonitrile-butadiene-styrene (ABS) [46]. It is also often used in wood fillers as the attached MSDS certifies as an example. In this case, 15–20% styrene is present, making the product flammable, harmful and irritating. Again, it is assumed that the starting raw material is not derived from virgin wood.

PAHs were also found for the organic fraction. They are compounds naturally present in petroleum or coal, and from which they can be produced due to incomplete combustion of other molecules. PAHs, in addition to fossil fuels, can also be released from the combustion of other substrates, such as waste, tobacco, wood and charcoal. In general, therefore, PAHs are generated by incomplete combustion of organic material and are released into the air bound to soot particles [46]. As a result of the analysis carried out, four PAHs, phenanthrene, fluoranthene, perylene and pyrene, were found in some samples, specifically in samples #4 (sawdust wood), #B (briquettes) and #C (briquettes). Perylene, found only in sample #B, is present on Earth in tar and particulate matter due to air pollution. It can also be found in fossil crinoids and tropical termite mounds, as well as in peat, in recent sedimentary rocks at the bottom of water bodies and in crude oil. There are debates about the possible origin of perylene from the degradation of wood by fungi [47]. In addition, perylene is the progenitor of dyes called rilenes [48] and possesses blue fluorescence. Together with its derivatives, it is used as a dopant to generate blue luminescence in OLED devices [49] and is not considered hazardous according to Directive 67/548/EEC [50]. Pyrene, on the other hand, found in samples #4 and #C, is a PAH consisting of four condensed benzene rings. It is formed during incomplete combustion of organic compounds. It is used in the production of dyes [17]. It has kidney and liver toxicity but is not declared carcinogenic (IARC Group 3). Phenanthrene, found in samples #4, #B and #C, is a polycyclic aromatic hydrocarbon (PAH) composed of three fused benzene rings (C_14_H_10_). It is practically insoluble in water, whereas it is soluble in ether, benzene and toluene. Phenanthrene is a residue from the combustion of various organic substances and is present in cigarette smoke. According to IARC, it is in class 3 (“not carcinogenic to humans”). Similar toxicological information pertains to fluoranthene, found in #4, #B and #C [51].

### 3.3. Cluster Analysis and Principal Component Analysis

**Figure 4:**
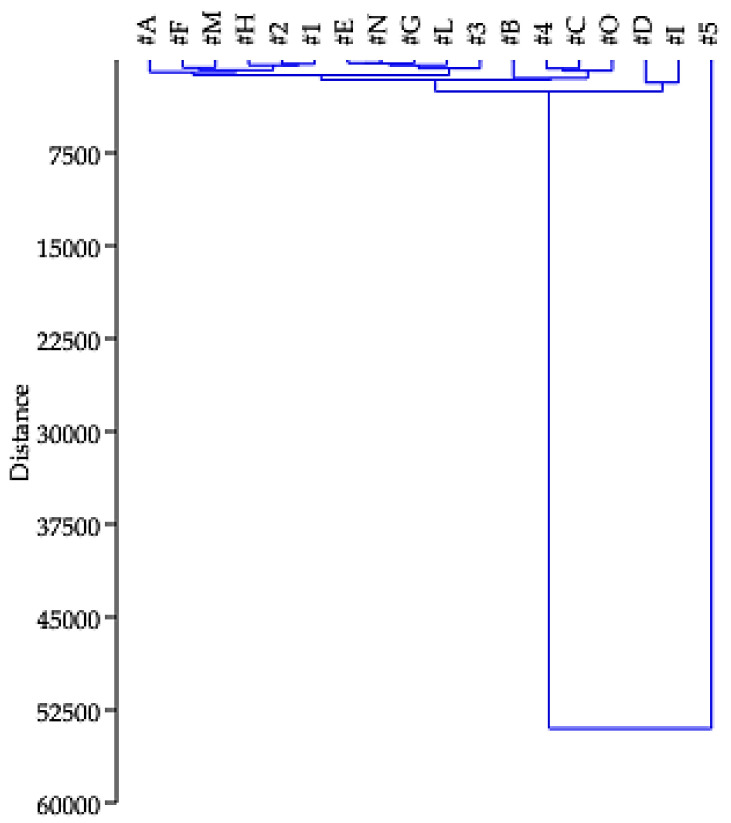
Dendogram displays the aggregation of pellet samples into five different clusters.

Table S2 shows some parameters of descriptive statistics, among which are standard deviation (SD) and variance (RDS). In contrast to the PAHs having low SD and RDS values, all other molecules found in the samples have standard deviations and variances that are not close to zero. This behavior is in line with the heterogeneity of the samples under analysis but needs to be further investigated using cluster analysis. Cluster analysis (CA) was performed on the dataset resulting from the analyses to verify the distribution of samples into similarity groups based on the analyzed chemical and physical variables [18]. CA was carried out using a hierarchical technique. The approach was carried out by means of Past software (version 4.11). Figure 4 shows the dendogram, which, on the basis of Euclidean distance, displays the aggregation of samples into groups. Based on the groupings, it is possible to determine the number of clusters in the order of 5. Specifically, the samples are distributed as follows: in cluster 1, samples #A, #F, #M, #H, #2 and #1 are grouped; in cluster 2, samples #E, #N, #G, #L and #3; in cluster 3, samples #B, #4, #C and #O; in cluster 4, samples #D and #I; in cluster 5, sample #5.

**Figure 5:**
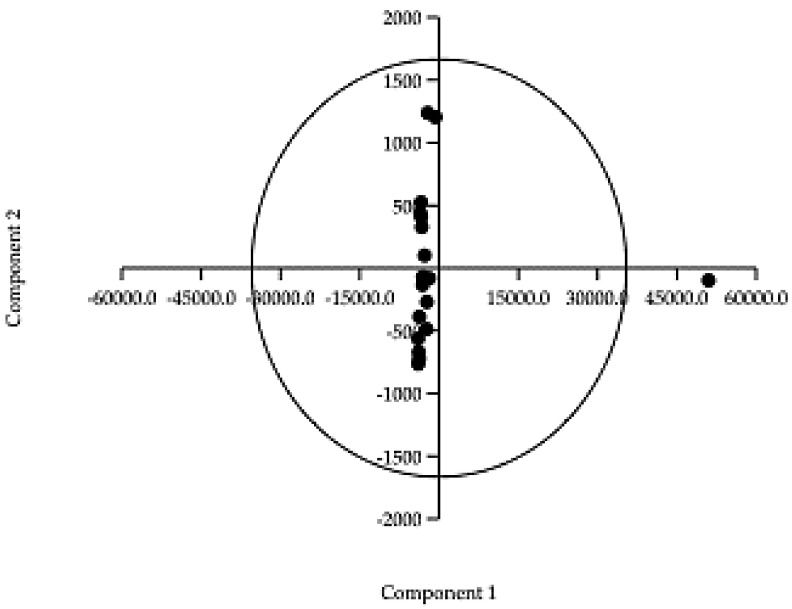
Principal component analysis (PCA) applied to all the samples. Component 1: Ca, Fe, Mg, K, Al, Na; Component 2: K, hydrocarbons, C_10_÷C_40_, Na, Fe.

Given the large number of parameters under study for the samples under consideration, principal component analysis (PCA) was carried out (using Past ver. 4.11) to graphically show the arrangement of the samples using a bidimensional plot. In fact, PCA allows the number of parameters to be reduced, usually to two or three, allowing a simplification of the database by generating new parameters called principal components (PCs) that equally represent the sample statistically. The choice of the number of PCs is based on the eigenvalue that expresses their statistical value; usually, those having a value greater than 1 are considered [52]. In this case, it would be sufficient to consider only one PC given the variance value achieved by the first PCs of 99.62%. As shown in Figure 5, all the samples are distributed along the PC1 axis (calcium, iron, magnesium, potassium, aluminum, sodium) except for sample #5, which instead presents totally different characteristics and by effect is distributed along the PC2 axis (potassium, hydrocarbons, C_10_ ÷ C_40_, sodium, iron), confirming its clear separation from the other samples already highlighted in the CA. In this case, we do not consider the hypothesis that it is an outlier given the clear diversity of the matrix analyzed compared to the other samples.

## 4. Discussion

Indoor and outdoor pollution related to airborne particles also pose health risks [53,54,55]. The use of pellets can pose health risks because of the indoor pollution they can generate. The first problem arises at the pellet storage stage. The best practice guide “Wood burning technologies for Irish consumers” of the Home Sustainable Energy Authority of Ireland (SEAI) recommends, for an average household, a stock of at least 10–15 kg bags of pellets. After several deaths experienced throughout Europe, the management and use of pellet storage space by ensuring adequate ventilation is critical [5]. It has been noted that pellets release carbon monoxide (CO) during the storage phase due to the oxidative degradation of fatty acids contained in the wood (more present in pine wood than spruce) [56,57,58], which is directly proportional to headspace, temperature and degree of pellet abrasion. CO levels can thus exceed the maximum exposure limit (447 ppm) [59]. Moisture can also slightly promote increased carbon monoxide release, but it is much more crucial, along with pellet chalking, in the onset of fungal infections by greatly increasing the risks of pellet dust inhalation due to the development of spores and toxins [5,47].

The use of pellets also promotes air pollution due to the production of inorganic pollutants such as heavy metals and organic pollutants such as benzene and derivatives. Inhalation of such compounds usually results in the increase in ROS within the body, which generate harmful effects in the body [60]. The analysis of heavy metals has reported interesting results. They are present in the environment due to both human and natural activities. Clearly, their amount has increased with industrial development, so urban and industrial areas are hot spots [34].

Lead, which represents the heavy metal with the greatest anthropogenic impact, can be taken up by humans through inhalation or ingestion by depositing in the lungs and entering the bloodstream [34]. The action of lead primarily damages the nervous system at any age. Pharmaceutical companies have set 1 μg g^−1^ as the maximum daily dose that can be taken [61].

The main route of the assimilation of nickel (Ni) by humans is through inhalation. A recent study quantified (1 mg Ni m^−3^) for soluble compounds and (10 mg Ni m^−3^) for mixtures of nickel compounds as the critical thresholds for the development of adverse effects [62]. Inhalation of this metal leads to an increase in ROS, which generates neurotoxicity and carcinogenicity. The IARC has placed nickel in Group 1 (human carcinogens) [21].

Ni and vanadium (V) can originate from fossil fuel use, and therefore a high presence of them in the environment is an indication of environmental pollution from these sources [63,64,65,66]. In 1960, Stocks highlighted the positive link between increased mortality as a result of respiratory, cardiac and cancer problems and the presence of V in the air. Exposure to V leads to respiratory problems; acute exposure brings visible effects from as low as 60 mg m^−3^, whereas chronic exposure occurs as early as 20 mg m^−3^, with symptoms that can range from cough to bronchitis and chronic pneumonia.

The concern about the presence of these metals following combustion is due to the cleanliness of boilers, where oxides of the relevant metals can be found in concentrations far higher than those of the source material [67,68]. Furthermore, studies have shown that the simultaneous presence of Ni and V is positively correlated with respiratory and cardiovascular problems, even at environmental exposure levels lower than the World Health Organization (WHO) standard for V (1 mg m^−3^ day^−1^) and the European Environment Agency (EEA) standard for Ni (1 mg m^−3^ day^−1^) [9].

The presence of formaldehyde in samples should not be underestimated. Considering that in some products used by the furniture industry, panels in particular, a resin derived from the condensation of urea with formaldehyde is used and that the emission of formaldehyde can be significant for long periods, some countries have imposed limitations for these materials. Formaldehyde is a substance whose potential danger is mainly linked to its extreme volatility. In fact, over time, its effects on human health have been studied, first noting irritation of the eyes and upper airways and, later, airway tumors, to the extent that it was classified in 2004 by the IARC as a human carcinogen (group 1) [69] and later by the European Commission as a carcinogen (category 1B) and mutagen (category 2). In 2010, the WHO listed 0.1 mg m^−3^ as the indoor guideline value for formaldehyde [70,71]. Formaldehyde is now considered a ubiquitous pollutant both indoors and outdoors. The main route of exposure for humans is inhalation. European scientific studies have determined the average formaldehyde concentrations to which the population is exposed, both outdoors (3–15 μg m^−3^) and indoors (20–40 μg m^−3^). For this very reason, the pellet storage area and its ventilation are crucial since air changes clearly influence the ambient concentration of formaldehyde [71].

With regard to the presence of toluene in some of the samples, this is of no particular concern given the levels found. Toluene is a neurotoxic molecule that is often used as an organic solvent or component of products such as adhesives and paint thinners. Because it is widely used in work environments, many countries have set occupational exposure limits (OELs) to avoid adverse health effects, which range from 14 to 300 mg kg^−1^ [72].

The presence of PAHs in samples can demonstrate areas of polluted raw material origin. Environmental PAH contamination is almost ubiquitous, but in areas of high anthropogenic impact, these increase significantly. Italy, especially the northern part, is one of the European countries that contributes the most to PAH emissions. Among the causes of the increasing emissions is domestic heating through the use of biomass, primarily wood, which is responsible for about 75% of PAH emissions [73]. The analysis performed showed the absence of benzo[a]pyrene, which is considered as a marker for the presence and effect of carcinogenic PAHs. The authors would like to underline that the BaP limit value in the atmosphere is 1 ng m^−3^ and that Directive 2004/107/EC [74] listed benzo(a)pyrene (BaP) as a marker of carcinogenic PAHs given its classification as a group 1 human carcinogen established by the IARC.

## 5. Conclusions

The introduction of pellets into the biofuel market had the aim of bringing benefits in terms of environmental emissions in those rural areas not reached by other energy sources other than coal or other more polluting sources. In other areas, however, the use of pellets has been encouraged as a green energy source as well as an alternative to wood-burning fireplaces used more as a means of furnishing than as a means of heating. Thus, together with green energy policies and the increase in the costs of other energy sources, wood fuels have seen a significant increase in the last two decades, even in developed countries. Despite being classified as biofuel, the use of pellets also generates environmental pollution problems, even if to a lesser extent than lignite briquettes and wood. As previously reported, the emissions from solid biofuel stoves are made up of both an inorganic and an organic fraction. The quantity and quality of the emissions depend on the type of stove, the type and quality of the fuel and the combustion parameters.

Given the results obtained from the analyses carried out, the authors of this work wish to emphasize the almost widespread presence of formaldehyde in the samples, an indication that the materials from which they originate certainly present sources of pollution. Thus, the Italian Pellet Gold certification, which implements the UNI EN ISO 17225 standard precisely by analyzing this pollutant, is important. However, the other organic substances found, which also pose a concern for human health, remain outside the parameters of this standard, even if found in smaller quantities, if we exclude sample #2 for acetone.

In recent years, Italian legislation has implemented regulations for the use of pellets. Many Italian regions have introduced limitations both on the use of pellet stoves and on the use of non-certified solid biofuels that are therefore potentially more polluting and harmful. For example, Lombardy, Piedmont, Veneto and Emilia-Romagna have signed an agreement for the improvement in air quality: in this agreement, there is an obligation to prohibit the installation of biofuel heat generators of class lower than four stars and the use of those with three stars by 31 December 2019 (decree 186/2017). This agreement requires the use of A1 certified pellets according to the UNI EN ISO 17225 standard. Therefore, in recent years, there has been an increasing trend towards regulating both the stoves and the quality of the pellets to be used.

The authors of this paper suggest increased regulation both to reduce air emissions and to protect people and the environment from potentially harmful chemicals from the source materials or their combustion. It is therefore important to constantly monitor the quality of the pellets and other biomass on the market in order to totally exclude those biomasses deriving from unauthorized waste products.

## Data Availability

The data presented in this study are available in the Supplementary Materials here.

## Supplementary Materials

The following supporting information can be downloaded at: https://www.mdpi.com/article/10.3390/ijerph20166559/s1, Table S1: Synoptic table of all the measurements carried out on the samples investigated in this study. LOD: limit of detection.; Table S2: Descriptive analytics of the measurements reported in Table S1. LOD: limit of detection.
