# Supplementary material for: Characteristics of Commercial and Raw Pellets Available on the Italian Market: Study of Organic and Inorganic Fraction and Related Chemometric Approach
Source: Int J Environ Res Public Health. 2023 Aug 11;20(16):6559. doi: 10.3390/ijerph20166559 (PMC10454322; doi:10.3390/ijerph20166559)
Supplement: Supplementary file 1 [file ijerph-20-06559-s001.zip › ijerph-2478825-supplementary.pdf]

Table S1. Synoptic table of all the measurements carried out on the samples investigated in this study. LOD: Limit of Detection.

**Analysis**	**Method**		**LOD**	**olive pomace (bulk) #1**	**olive pomace (bulk) #2**	**wood sawdust #3**	**wood sawdust #4**	**wood chips (pruning and wood waste) #5**	**logs (wood briquettes) #A**	**briquettes (wood briquettes) #B**	**briquettes (wood briquettes) #C**	**briquettes (wood briquettes) #D**	**Canadian pellets #E**	**Romanian pellets #F**	**pellets #G**	**pomace peanuts #H**	**briquettes (pomace briquettes) #I**	**Romanian pellet #L**	**Romanian pellet #M**	**pellet #N**	**pellet #O**
Physical state	visual	-	-	solid	solid	solid	solid	solid	solid	solid	solid	solid	solid	solid	solid	solid	solid	solid	solid	solid	solid
Color	visual	-	-	dark brown	dark brown	light brown	light brown	dark brown	light brown	light brown	light brown	brown	brown	brown	light brown	light brown	dark brown	light brown	brown	brown	light brown
Odor	olfactory	-	-	pom-ace	pom-ace	wood	wood	vege-table	wood	wood	wood	wood	pellet	pellet	pellet	pomace	pomace	pellet	pellet	pellet	pellet
pH	CNR IRSA 1 Q64 Vol. 3 1985	pH unit	-	3.7	4.1	4.2	5.2	7.8	4.8	5.5	5	4.7	4	5	4.4	3.9	4.6	4.1	5.2	4.1	5.6
Dry matter at 105 °C	CNR IRSA 2 Q64 Vol. 2 1984	% w/w	-	82.5	82.4	90.5	87.5	53.7	90.7	90.7	89.3	90	93.3	95.7	92.7	82.8	88.9	91.8	94	94.2	95.7
Humidity	CNR IRSA 2 Q64 Vol. 2 1984	% w/w	-	17.5	17.6	9.5	12.5	46.3	9.3	9.3	10.7	10	6.7	4.3	7.3	17.2	11.1	8.2	6	5.8	4.3
Dry matter at 600 °C	CNR IRSA 2 Q64 Vol. 2 1985	% w/w	-	0.79	0.87	0.33	1.1	4	1.1	2	1.2	2.4	0.36	0.97	0.32	0.81	3	0.39	0.92	0.38	0.95
Total Organic Compound (TOC)	UNI EN 13137:2002	% w/w	-	45.8	42.3	46.2	43.5	17.8	40.9	41.6	44.5	45.3	47.7	47.2	46.1	41.4	46.6	47.1	46.2	47.3	47.3

Sulfur	CNR IRSA 12 Q64 Vol. 3 1986	mg/kg	10	<10	<10	<10	<10	<10	<10	<10	<10	<10	<10	<10	<10	<10	<10	<10	<10	<10	<10
Total cyanides	EPA 9013A 2004 + EPA 9010C 2004 + EPA 9014 1996	mg/kg	0.1	<0.1	<0.1	<0.1	<0.1	<0.1	<0.1	<0.1	<0.1	<0.1	<0.1	<0.1	<0.1	<0.1	<0.1	<0.1	<0.1	<0.1	<0.1
Metals																					
- Aluminum	UNI EN 13657:2004 + EPA 6010C 2007	mg/kg		21.2	19.8	2.5	35.5	2540	15.2	78.4	66.6	45.7	24.9	34.5	7.7	17.8	45	10.7	31.3	19.9	53.3
- Antimony	UNI EN 13657:2004 + EPA 6010C 2007	mg/kg	0.5	<0.5	<0.5	<0.5	<0.5	0.78	<0.5	<0.5	<0.5	<0.5	<0.5	<0.5	<0.5	<0.5	<0.5	<0.5	<0.5	<0.5	<0.5
- Silver	UNI EN 13657:2004 + EPA 6010C 2007	mg/kg	0.5	<0.5	<0.5	<0.5	<0.5	<0.5	<0.5	<0.5	<0.5	<0.5	<0.5	<0.5	<0.5	<0.5	<0.5	<0.5	<0.5	<0.5	<0.5
- Arsenic	UNI EN 13657:2004 + EPA 6010C 2007	mg/kg	0.5	<0.5	<0.5	<0.5	<0.5	2.3	<0.5	<0.5	<0.5	<0.5	<0.5	<0.5	<0.5	<0.5	<0.5	<0.5	<0.5	<0.5	<0.5
- Barium	UNI EN 13657:2004 + EPA 6010C 2007	mg/kg		1	1.2	3.8	15.6	55.6	3.7	22.4	34.4	7.1	5.8	29.7	10.1	0.86	5.4	12.4	30.1	5	31.1
- Beryllium	UNI EN 13657:2004 + EPA 6010C 2007	mg/kg	0.5	<0.5	<0.5	<0.5	<0.5	<0.5	<0.5	<0.5	<0.5	<0.5	<0.5	<0.5	<0.5	<0.5	<0.5	<0.5	<0.5	<0.5	<0.5
- Boron	UNI EN 13657:2004 + EPA 6010C 2007	mg/kg		5.7	5.8	6.1	5.9	64.7	4.6	4	9.7	8.3	1.6	3.1	7.5	5.2	8.4	1.7	2.3	1.7	3.6

- Cadmium	UNI EN 13657:2004 + EPA 6010C 2007	mg/kg	0.25	<0.25	<0.25	<0.25	<0.25	0.34	<0.25	<0.25	<0.25	<0.25	<0.25	<0.25	<0.25	<0.25	<0.25	<0.25	<0.25	<0.25	<0.25
- Calcium	UNI EN 13657:2004 + EPA 6010C 2007	mg/kg		1095	1225	810	2180	54960	935	1820	2665	2225	585	1430	815	1115	3680	745	1395	635	2200
- Cobalt	UNI EN 13657:2004 + EPA 6010C 2007	mg/kg	0.5	<0.5	<0.5	<0.5	<0.5	2.7	<0.5	<0.5	<0.5	<0.5	<0.5	<0.5	<0.5	<0.5	<0.5	<0.5	<0.5	<0.5	<0.5
- Total Chromium	UNI EN 13657:2004 + EPA 6010C 2007	mg/kg	0.5	<0.5	1.1	<0.5	0.57	26.6	<0.5	0.91	1.5	2.6	<0.5	<0.5	<0.5	<0.5	0.89	<0.5	<0.5	<0.5	<0.5
- Chromium VI	CNR IRSA 16 Q64 Vol. 3 1986	mg/kg	1	<1.0	<1.0	<1.0	<1.0	<1.0	<1.0	<1.0	<1.0	<1.0	<1.0	<1.0	<1.0	<1.0	<1.0	<1.0	<1.0	<1.0	<1.0
- Iron	UNI EN 13657:2004 + EPA 6010C 2007	mg/kg		51.8	59.2	6.9	90.5	4655	33	81	122	1135	42.7	73.2	13.9	89.9	250	66.3	74.7	15.3	81.1
- Magnesium	UNI EN 13657:2004 + EPA 6010C 2007	mg/kg		116	137	182	225	4275	212	190	340	343	155	230	95.5	123	465	95.2	305	225	370
- Manganese	UNI EN 13657:2004 + EPA 6010C 2007	mg/kg		3.2	3.7	8.8	19	260	7.4	24.7	31.9	37.7	60.9	69.8	80.8	3.4	20.5	93.6	56.8	25.1	100
- Mercury	UNI EN 13657:2004 + EPA 6010C 2007	mg/kg	0.5	<0.5	<0.5	<0.5	<0.5	<0.5	<0.5	<0.5	<0.5	<0.5	<0.5	<0.5	<0.5	<0.5	<0.5	<0.5	<0.5	<0.5	<0.5
- Molybdenium	UNI EN 13657:2004 +	mg/kg	0.5	<0.5	<0.5	<0.5	<0.5	0.94	<0.5	<0.5	<0.5	<0.5	<0.5	<0.5	<0.5	<0.5	<0.5	<0.5	<0.5	<0.5	<0.5

	EPA 6010C 2007																				
- Nickel	UNI EN 13657:2004 + EPA 6010C 2007	mg/kg	0.5	<0.5	<0.5	<0.5	<0.5	13.2	<0.5	<0.5	<0.5	1	<0.5	0.54	<0.5	<0.5	0.59	<0.5	<0.5	<0.5	<0.5
- Lead	UNI EN 13657:2004 + EPA 6010C 2007	mg/kg	0.5	<0.5	<0.5	<0.5	4.6	11.5	<0.5	6.9	6	1.4	<0.5	1.2	<0.5	<0.5	0.7	<0.5	0.67	<0.5	0.72
- Potassium	UNI EN 13657:2004 + EPA 6010C 2007	mg/kg		1540	1425	825	565	4780	1080	590	880	1800	415	795	295	1620	2280	152	1010	235	1100
- Copper	UNI EN 13657:2004 + EPA 6010C 2007	mg/kg		2.3	2.9	1	1.9	24.9	1.3	1.9	3.3	4.4	0.72	0.94	<0.5	2.1	5.3	0.6	0.97	0.53	1.1
- Selenium	UNI EN 13657:2004 + EPA 6010C 2007	mg/kg	0.5	<0.5	<0.5	<0.5	<0.5	<0.5	<0.5	<0.5	<0.5	<0.5	<0.5	<0.5	<0.5	<0.5	<0.5	<0.5	<0.5	<0.5	<0.5
- Silicon	UNI EN 13657:2004 + EPA 6010C 2007	mg/kg		36	51.4	4.8	48.9	64.8	15.3	25.8	70	59.7	14.4	52.1	14.1	37.6	63.7	17.5	46.6	22.9	63.9
- Sodium	UNI EN 13657:2004 + EPA 6010C 2007	mg/kg		67.1	97.2	45.4	75.6	1145	190	96	130	530	22.9	68.52	13.7	93.3	710	20.4	21.1	49.4	8.8
- Tin	UNI EN 13657:2004 + EPA 6010C 2007	mg/kg	0.5	<0.5	<0.5	<0.5	<0.5	0.69	<0.5	<0.5	<0.5	<0.5	<0.5	<0.5	<0.5	<0.5	<0.5	<0.5	<0.5	<0.5	<0.5
- Thallium	UNI EN 13657:2004 + EPA 6010C 2007	mg/kg	0.5	<0.5	<0.5	<0.5	<0.5	<0.5	<0.5	<0.5	<0.5	<0.5	<0.5	<0.5	<0.5	<0.5	<0.5	<0.5	<0.5	<0.5	<0.5

- Tellurium	UNI EN 13657:2004 + EPA 6010C 2007	mg/kg	0.5	<0.5	<0.5	<0.5	<0.5	<0.5	<0.5	<0.5	<0.5	<0.5	<0.5	<0.5	<0.5	<0.5	<0.5	<0.5	<0.5	<0.5	<0.5
- Titanium	UNI EN 13657:2004 + EPA 6010C 2007	mg/kg	0.5	<0.5	0.74	<0.5	4.6	50.3	<0.5	4.2	7.6	2	<0.5	<0.5	<0.5	<0.5	1.2	0.67	0.82	<0.5	1.7
- Vanadium	UNI EN 13657:2004 + EPA 6010C 2007	mg/kg	0.5	0.66	0.64	0.69	0.64	8.9	0.62	0.6	0.82	0.88	<0.5	0.66	<0.5	0.62	0.81	0.73	0.62	<0.5	0.72
- Zinc	UNI EN 13657:2004 + EPA 6010C 2007	mg/kg		1.3	1.6	1.6	9.1	58.2	3	17	15.1	11.4	5.6	4.6	5.9	1.8	9.2	6	3.8	5.1	3.5
Aliphatic hydrocarbons (C5÷C8)	EPA 5035A 2002 + EPA 8015D 2003	mg/kg	10	<10	<10	<10	<10	<10	<10	<10	<10	<10	<10	<10	<10	<10	<10	<10	<10	<10	<10
Aromatic hydrocarbons (C9÷C10)	EPA 5035A 2002 + EPA 8015D 2003	mg/kg	10	<10	<10	<10	<10	<10	<10	<10	<10	<10	<10	<10	<10	<10	<10	<10	<10	<10	<10
Hydrocarbons (C10÷C40)	UNI EN 14039:2005	mg/kg		830	605	<100	570	940	1410	1770	935	1435	435	945	365	390	1015	710	415	325	<100
Aromatic organic compounds																					-
- Benzene	EPA 5035A 2002 + EPA 8260C 2006	mg/kg	0.1	<0.1	<0.1	<0.1	<0.1	<0.1	<0.1	<0.1	<0.1	<0.1	<0.1	<0.1	<0.1	<0.1	<0.1	<0.1	<0.1	<0.1	<0.1
- Toluene	EPA 5035A 2002 + EPA 8260C 2006	mg/kg	0.1	<0.1	<0.1	<0.1	<0.1	<0.1	<0.1	1.2	0.23	<0.1	<0.1	<0.1	0.6	<0.1	<0.1	<0.1	<0.1	<0.1	<0.1
- Ethylbenzene	EPA 5035A 2002 + EPA 8260C 2006	mg/kg	0.1	<0.1	<0.1	<0.1	<0.1	<0.1	<0.1	<0.1	<0.1	<0.1	<0.1	<0.1	<0.1	<0.1	<0.1	<0.1	<0.1	<0.1	<0.1

- o-Xylene	EPA 5035A 2002 + EPA 8260C 2006	mg/kg	0.1	<0.1	<0.1	<0.1	<0.1	<0.1	<0.1	<0.1	<0.1	<0.1	<0.1	<0.1	<0.1	<0.1	<0.1	<0.1	<0.1	<0.1	<0.1
- m-Xylene	EPA 5035A 2002 + EPA 8260C 2006	mg/kg	0.1	<0.1	<0.1	<0.1	<0.1	<0.1	<0.1	<0.1	<0.1	<0.1	<0.1	<0.1	<0.1	<0.1	<0.1	<0.1	<0.1	<0.1	<0.1
- p-Xylene	EPA 5035A 2002 + EPA 8260C 2006	mg/kg	0.1	<0.1	<0.1	<0.1	<0.1	<0.1	<0.1	<0.1	<0.1	<0.1	<0.1	<0.1	<0.1	<0.1	<0.1	<0.1	<0.1	<0.1	<0.1
- Styrene	EPA 5035A 2002 + EPA 8260C 2006	mg/kg	0.1	<0.1	<0.1	<0.1	<0.1	<0.1	<0.1	0.57	<0.1	<0.1	<0.1	<0.1	<0.1	<0.1	<0.1	<0.1	<0.1	<0.1	<0.1
- Isopropylbenzene	EPA 5035A 2002 + EPA 8260C 2006	mg/kg	0.1	<0.1	<0.1	<0.1	<0.1	<0.1	<0.1	<0.1	<0.1	<0.1	<0.1	<0.1	<0.1	<0.1	<0.1	<0.1	<0.1	<0.1	<0.1
- 1,2,4-Trimethylbenzene	EPA 5035A 2002 + EPA 8260C 2006	mg/kg	0.1	<0.1	<0.1	<0.1	<0.1	<0.1	<0.1	<0.1	<0.1	<0.1	<0.1	<0.1	<0.1	<0.1	<0.1	<0.1	<0.1	<0.1	<0.1
- 1.3.5-Trimethylbenzene	EPA 5035A 2002 + EPA 8260C 2006	mg/kg	0.1	<0.1	<0.1	<0.1	<0.1	<0.1	<0.1	<0.1	<0.1	<0.1	<0.1	<0.1	<0.1	<0.1	<0.1	<0.1	<0.1	<0.1	<0.1
Chlorinated Aliphatic Compounds																					-
- Chloromethane	EPA 5035A 2002 + EPA 8260C 2006	mg/kg	0.1	<0.1	<0.1	<0.1	<0.1	<0.1	<0.1	<0.1	<0.1	<0.1	<0.1	<0.1	<0.1	<0.1	<0.1	<0.1	<0.1	<0.1	<0.1
- Vinyl Chloride	EPA 5035A 2002 + EPA 8260C 2006	mg/kg	0.1	<0.1	<0.1	<0.1	<0.1	<0.1	<0.1	<0.1	<0.1	<0.1	<0.1	<0.1	<0.1	<0.1	<0.1	<0.1	<0.1	<0.1	<0.1
- 1.1-Dichloroethylene	EPA 5035A 2002 + EPA 8260C 2006	mg/kg	0.1	<0.1	<0.1	<0.1	<0.1	<0.1	<0.1	<0.1	<0.1	<0.1	<0.1	<0.1	<0.1	<0.1	<0.1	<0.1	<0.1	<0.1	<0.1
- Dichloromethane	EPA 5035A 2002 + EPA 8260C 2006	mg/kg	0.1	<0.1	<0.1	<0.1	<0.1	<0.1	<0.1	<0.1	<0.1	<0.1	<0.1	<0.1	<0.1	<0.1	<0.1	<0.1	<0.1	<0.1	<0.1

- trans-1.2-Dichloroethylene	EPA 5035A 2002 + EPA 8260C 2006	mg/kg	0.1	<0.1	<0.1	<0.1	<0.1	<0.1	<0.1	<0.1	<0.1	<0.1	<0.1	<0.1	<0.1	<0.1	<0.1	<0.1	<0.1	<0.1	<0.1
- 1.1-Dichloroethane	EPA 5035A 2002 + EPA 8260C 2006	mg/kg	0.1	<0.1	<0.1	<0.1	<0.1	<0.1	<0.1	<0.1	<0.1	<0.1	<0.1	<0.1	<0.1	<0.1	<0.1	<0.1	<0.1	<0.1	<0.1
- Trichloromethane (Chloroform)	EPA 5035A 2002 + EPA 8260C 2006	mg/kg	0.1	<0.1	<0.1	<0.1	<0.1	<0.1	<0.1	<0.1	<0.1	<0.1	<0.1	<0.1	<0.1	<0.1	<0.1	<0.1	<0.1	<0.1	<0.1
- cis-1.2-Dichloroethylene	EPA 5035A 2002 + EPA 8260C 2006	mg/kg	0.1	<0.1	<0.1	<0.1	<0.1	<0.1	<0.1	<0.1	<0.1	<0.1	<0.1	<0.1	<0.1	<0.1	<0.1	<0.1	<0.1	<0.1	<0.1
- 1.1.1-Trichloroethane	EPA 5035A 2002 + EPA 8260C 2006	mg/kg	0.1	<0.1	<0.1	<0.1	<0.1	<0.1	<0.1	<0.1	<0.1	<0.1	<0.1	<0.1	<0.1	<0.1	<0.1	<0.1	<0.1	<0.1	<0.1
- Carbon tetrachloride	EPA 5035A 2002 + EPA 8260C 2006	mg/kg	0.1	<0.1	<0.1	<0.1	<0.1	<0.1	<0.1	<0.1	<0.1	<0.1	<0.1	<0.1	<0.1	<0.1	<0.1	<0.1	<0.1	<0.1	<0.1
- 1.1-Dichloropropene	EPA 5035A 2002 + EPA 8260C 2006	mg/kg	0.1	<0.1	<0.1	<0.1	<0.1	<0.1	<0.1	<0.1	<0.1	<0.1	<0.1	<0.1	<0.1	<0.1	<0.1	<0.1	<0.1	<0.1	<0.1
- Trichloroethylene	EPA 5035A 2002 + EPA 8260C 2006	mg/kg	0.1	<0.1	<0.1	<0.1	<0.1	<0.1	<0.1	<0.1	<0.1	<0.1	<0.1	<0.1	<0.1	<0.1	<0.1	<0.1	<0.1	<0.1	<0.1
- 1.2-Dichloroethane	EPA 5035A 2002 + EPA 8260C 2006	mg/kg	0.1	<0.1	<0.1	<0.1	<0.1	<0.1	<0.1	<0.1	<0.1	<0.1	<0.1	<0.1	<0.1	<0.1	<0.1	<0.1	<0.1	<0.1	<0.1
- 1.2-Dichloropropane	EPA 5035A 2002 + EPA 8260C 2006	mg/kg	0.1	<0.1	<0.1	<0.1	<0.1	<0.1	<0.1	<0.1	<0.1	<0.1	<0.1	<0.1	<0.1	<0.1	<0.1	<0.1	<0.1	<0.1	<0.1
- cis-1.3-Dichloropropene	EPA 5035A 2002 + EPA 8260C 2006	mg/kg	0.1	<0.1	<0.1	<0.1	<0.1	<0.1	<0.1	<0.1	<0.1	<0.1	<0.1	<0.1	<0.1	<0.1	<0.1	<0.1	<0.1	<0.1	<0.1
- Tetrachloroethylene (PCE)	EPA 5035A 2002 + EPA 8260C 2006	mg/kg	0.1	<0.1	<0.1	<0.1	<0.1	<0.1	<0.1	<0.1	<0.1	<0.1	<0.1	<0.1	<0.1	<0.1	<0.1	<0.1	<0.1	<0.1	<0.1

- 1.1.2-Trichloroethane	EPA 5035A 2002 + EPA 8260C 2006	mg/kg	0.1	<0.1	<0.1	<0.1	<0.1	<0.1	<0.1	<0.1	<0.1	<0.1	<0.1	<0.1	<0.1	<0.1	<0.1	<0.1	<0.1	<0.1	<0.1
- trans-1.3-Dichloropropene	EPA 5035A 2002 + EPA 8260C 2006	mg/kg	0.1	<0.1	<0.1	<0.1	<0.1	<0.1	<0.1	<0.1	<0.1	<0.1	<0.1	<0.1	<0.1	<0.1	<0.1	<0.1	<0.1	<0.1	<0.1
- 1.1.1.2-Tetrachloroethane	EPA 5035A 2002 + EPA 8260C 2006	mg/kg	0.1	<0.1	<0.1	<0.1	<0.1	<0.1	<0.1	<0.1	<0.1	<0.1	<0.1	<0.1	<0.1	<0.1	<0.1	<0.1	<0.1	<0.1	<0.1
- 1.3-Dichloropropane	EPA 5035A 2002 + EPA 8260C 2006	mg/kg	0.1	<0.1	<0.1	<0.1	<0.1	<0.1	<0.1	<0.1	<0.1	<0.1	<0.1	<0.1	<0.1	<0.1	<0.1	<0.1	<0.1	<0.1	<0.1
- 1.1.2.2-Tetrachloroethane	EPA 5035A 2002 + EPA 8260C 2006	mg/kg	0.1	<0.1	<0.1	<0.1	<0.1	<0.1	<0.1	<0.1	<0.1	<0.1	<0.1	<0.1	<0.1	<0.1	<0.1	<0.1	<0.1	<0.1	<0.1
- 1.2.3-Trichloropropane	EPA 5035A 2002 + EPA 8260C 2006	mg/kg	0.1	<0.1	<0.1	<0.1	<0.1	<0.1	<0.1	<0.1	<0.1	<0.1	<0.1	<0.1	<0.1	<0.1	<0.1	<0.1	<0.1	<0.1	<0.1
- Hexachlorobutadiene	EPA 5035A 2002 + EPA 8260C 2006	mg/kg	0.1	<0.1	<0.1	<0.1	<0.1	<0.1	<0.1	<0.1	<0.1	<0.1	<0.1	<0.1	<0.1	<0.1	<0.1	<0.1	<0.1	<0.1	<0.1
Aliphatic Halogenated Compounds																					-
- 1.2-Dibromo-3-chloropropane	EPA 5035A 2002 + EPA 8260C 2006	mg/kg	0.1	<0.1	<0.1	<0.1	<0.1	<0.1	<0.1	<0.1	<0.1	<0.1	<0.1	<0.1	<0.1	<0.1	<0.1	<0.1	<0.1	<0.1	<0.1
- Bromochloromethane	EPA 5035A 2002 + EPA 8260C 2006	mg/kg	0.1	<0.1	<0.1	<0.1	<0.1	<0.1	<0.1	<0.1	<0.1	<0.1	<0.1	<0.1	<0.1	<0.1	<0.1	<0.1	<0.1	<0.1	<0.1
- Bromodichloromethane	EPA 5035A 2002 + EPA 8260C 2006	mg/kg	0.1	<0.1	<0.1	<0.1	<0.1	<0.1	<0.1	<0.1	<0.1	<0.1	<0.1	<0.1	<0.1	<0.1	<0.1	<0.1	<0.1	<0.1	<0.1
- Dibromochloromethane	EPA 5035A 2002 + EPA 8260C 2006	mg/kg	0.1	<0.1	<0.1	<0.1	<0.1	<0.1	<0.1	<0.1	<0.1	<0.1	<0.1	<0.1	<0.1	<0.1	<0.1	<0.1	<0.1	<0.1	<0.1

- 1.2-Dibromo-ethane	EPA 5035A 2002 + EPA 8260C 2006	mg/kg	0.1	<0.1	<0.1	<0.1	<0.1	<0.1	<0.1	<0.1	<0.1	<0.1	<0.1	<0.1	<0.1	<0.1	<0.1	<0.1	<0.1	<0.1	<0.1
- Tribromo-methane (Bro-moform)	EPA 5035A 2002 + EPA 8260C 2006	mg/kg	0.1	<0.1	<0.1	<0.1	<0.1	<0.1	<0.1	<0.1	<0.1	<0.1	<0.1	<0.1	<0.1	<0.1	<0.1	<0.1	<0.1	<0.1	<0.1
Polycyclic Aro-matic Hydro-carbons																					
- Naphthalene	EPA 3550C 2007 + EPA 8270D 2007	mg/kg	0.1	<0.1	<0.1	<0.1	<0.1	<0.1	<0.1	<0.1	<0.1	<0.1	<0.1	<0.1	<0.1	<0.1	<0.1	<0.1	<0.1	<0.1	<0.1
- Acenaphtilene	EPA 3550C 2007 + EPA 8270D 2007	mg/kg	0.1	<0.1	<0.1	<0.1	<0.1	<0.1	<0.1	<0.1	<0.1	<0.1	<0.1	<0.1	<0.1	<0.1	<0.1	<0.1	<0.1	<0.1	<0.1
- Acenaftene	EPA 3550C 2007 + EPA 8270D 2007	mg/kg	0.1	<0.1	<0.1	<0.1	<0.1	<0.1	<0.1	<0.1	<0.1	<0.1	<0.1	<0.1	<0.1	<0.1	<0.1	<0.1	<0.1	<0.1	<0.1
- Fluorenes	EPA 3550C 2007 + EPA 8270D 2007	mg/kg	0.1	<0.1	<0.1	<0.1	<0.1	<0.1	<0.1	<0.1	<0.1	<0.1	<0.1	<0.1	<0.1	<0.1	<0.1	<0.1	<0.1	<0.1	<0.1
- Fenanthrene	EPA 3550C 2007 + EPA 8270D 2007	mg/kg	0.1	<0.1	<0.1	<0.1	0.23	<0.1	<0.1	0.34	0.23	<0.1	<0.1	<0.1	<0.1	<0.1	<0.1	<0.1	<0.1	<0.1	<0.1
- Anthracene	EPA 3550C 2007 + EPA 8270D 2007	mg/kg	0.1	<0.1	<0.1	<0.1	<0.1	<0.1	<0.1	<0.1	<0.1	<0.1	<0.1	<0.1	<0.1	<0.1	<0.1	<0.1	<0.1	<0.1	<0.1
- Fluorantenes	EPA 3550C 2007 + EPA 8270D 2007	mg/kg	0.1	<0.1	<0.1	<0.1	0.16	<0.1	<0.1	0.25	0.2	<0.1	<0.1	<0.1	<0.1	<0.1	<0.1	<0.1	<0.1	<0.1	<0.1
- Perylene	EPA 3550C 2007 + EPA 8270D 2007	mg/kg	0.1	<0.1	<0.1	<0.1	<0.1	<0.1	<0.1	0.16	<0.1	<0.1	<0.1	<0.1	<0.1	<0.1	<0.1	<0.1	<0.1	<0.1	<0.1
- Pyrene	EPA 3550C 2007 + EPA 8270D 2007	mg/kg	0.1	<0.1	<0.1	<0.1	0.12	<0.1	<0.1	<0.1	0.14	<0.1	<0.1	<0.1	<0.1	<0.1	<0.1	<0.1	<0.1	<0.1	<0.1

- Benzo(a)anthracene	EPA 3550C 2007 + EPA 8270D 2007	mg/kg	0.1	<0.1	<0.1	<0.1	<0.1	<0.1	<0.1	<0.1	<0.1	<0.1	<0.1	<0.1	<0.1	<0.1	<0.1	<0.1	<0.1	<0.1	<0.1
- Crysene	EPA 3550C 2007 + EPA 8270D 2007	mg/kg	0.1	<0.1	<0.1	<0.1	<0.1	<0.1	<0.1	<0.1	<0.1	<0.1	<0.1	<0.1	<0.1	<0.1	<0.1	<0.1	<0.1	<0.1	<0.1
- Benzo(b)fluorantenes	EPA 3550C 2007 + EPA 8270D 2007	mg/kg	0.1	<0.1	<0.1	<0.1	<0.1	<0.1	<0.1	<0.1	<0.1	<0.1	<0.1	<0.1	<0.1	<0.1	<0.1	<0.1	<0.1	<0.1	<0.1
- Benzo(k+j)fluorantenes	EPA 3550C 2007 + EPA 8270D 2007	mg/kg	0.2	<0.2	<0.2	<0.2	<0.2	<0.2	<0.2	<0.2	<0.2	<0.2	<0.2	<0.2	<0.2	<0.2	<0.2	<0.2	<0.2	<0.2	<0.2
- Benzo(e)pyrene	EPA 3550C 2007 + EPA 8270D 2007	mg/kg	0.1	<0.1	<0.1	<0.1	<0.1	<0.1	<0.1	<0.1	<0.1	<0.1	<0.1	<0.1	<0.1	<0.1	<0.1	<0.1	<0.1	<0.1	<0.1
- Benzo(a)pyrene	EPA 3550C 2007 + EPA 8270D 2007	mg/kg	0.1	<0.1	<0.1	<0.1	<0.1	<0.1	<0.1	<0.1	<0.1	<0.1	<0.1	<0.1	<0.1	<0.1	<0.1	<0.1	<0.1	<0.1	<0.1
- Indeno(1.2.3-cd)pyrenees	EPA 3550C 2007 + EPA 8270D 2007	mg/kg	0.1	<0.1	<0.1	<0.1	<0.1	<0.1	<0.1	<0.1	<0.1	<0.1	<0.1	<0.1	<0.1	<0.1	<0.1	<0.1	<0.1	<0.1	<0.1
- Dibenzo(ah)anthracene	EPA 3550C 2007 + EPA 8270D 2007	mg/kg	0.1	<0.1	<0.1	<0.1	<0.1	<0.1	<0.1	<0.1	<0.1	<0.1	<0.1	<0.1	<0.1	<0.1	<0.1	<0.1	<0.1	<0.1	<0.1
- Benzo(ghi)perilene	EPA 3550C 2007 + EPA 8270D 2007	mg/kg	0.1	<0.1	<0.1	<0.1	<0.1	<0.1	<0.1	<0.1	<0.1	<0.1	<0.1	<0.1	<0.1	<0.1	<0.1	<0.1	<0.1	<0.1	<0.1
- Dibenzo(al)pyrene	EPA 3550C 2007 + EPA 8270D 2007	mg/kg	0.1	<0.1	<0.1	<0.1	<0.1	<0.1	<0.1	<0.1	<0.1	<0.1	<0.1	<0.1	<0.1	<0.1	<0.1	<0.1	<0.1	<0.1	<0.1
- Dibenzo(ae)pyrene	EPA 3550C 2007 + EPA 8270D 2007	mg/kg	0.1	<0.1	<0.1	<0.1	<0.1	<0.1	<0.1	<0.1	<0.1	<0.1	<0.1	<0.1	<0.1	<0.1	<0.1	<0.1	<0.1	<0.1	<0.1
- Dibenzo(ai)pyrene	EPA 3550C 2007 + EPA 8270D 2007	mg/kg	0.1	<0.1	<0.1	<0.1	<0.1	<0.1	<0.1	<0.1	<0.1	<0.1	<0.1	<0.1	<0.1	<0.1	<0.1	<0.1	<0.1	<0.1	<0.1

- Dibenzo(ah)pyrene	EPA 3550C 2007 + EPA 8270D 2007	mg/kg	0..1	<0.1	<0.1	<0.1	<0.1	<0.1	<0.1	<0.1	<0.1	<0.1	<0.1	<0.1	<0.1	<0.1	<0.1	<0.1	<0.1	<0.1	<0.1
Chlorinated pesticides																					
- Etridiazole	EPA 3550C 2007 + EPA 8270D 2007	mg/kg	0.1	<0.1	<0.1	<0.1	<0.1	<0.1	<0.1	<0.1	<0.1	<0.1	<0.1	<0.1	<0.1	<0.1	<0.1	<0.1	<0.1	<0.1	<0.1
- Chloroneb	EPA 3550C 2007 + EPA 8270D 2007	mg/kg	0.1	<0.1	<0.1	<0.1	<0.1	<0.1	<0.1	<0.1	<0.1	<0.1	<0.1	<0.1	<0.1	<0.1	<0.1	<0.1	<0.1	<0.1	<0.1
- alfa-BHC	EPA 3550C 2007 + EPA 8270D 2007	mg/kg	0.1	<0.1	<0.1	<0.1	<0.1	<0.1	<0.1	<0.1	<0.1	<0.1	<0.1	<0.1	<0.1	<0.1	<0.1	<0.1	<0.1	<0.1	<0.1
- Simazine	EPA 3550C 2007 + EPA 8270D 2007	mg/kg	0.1	<0.1	<0.1	<0.1	<0.1	<0.1	<0.1	<0.1	<0.1	<0.1	<0.1	<0.1	<0.1	<0.1	<0.1	<0.1	<0.1	<0.1	<0.1
- Atrazine	EPA 3550C 2007 + EPA 8270D 2007	mg/kg	0.1	<0.1	<0.1	<0.1	<0.1	<0.1	<0.1	<0.1	<0.1	<0.1	<0.1	<0.1	<0.1	<0.1	<0.1	<0.1	<0.1	<0.1	<0.1
- beta-BHC	EPA 3550C 2007 + EPA 8270D 2007	mg/kg	0.1	<0.1	<0.1	<0.1	<0.1	<0.1	<0.1	<0.1	<0.1	<0.1	<0.1	<0.1	<0.1	<0.1	<0.1	<0.1	<0.1	<0.1	<0.1
- gamma-BHC (Lindane)	EPA 3550C 2007 + EPA 8270D 2007	mg/kg	0.1	<0.1	<0.1	<0.1	<0.1	<0.1	<0.1	<0.1	<0.1	<0.1	<0.1	<0.1	<0.1	<0.1	<0.1	<0.1	<0.1	<0.1	<0.1
- delta-BHC	EPA 3550C 2007 + EPA 8270D 2007	mg/kg	0.1	<0.1	<0.1	<0.1	<0.1	<0.1	<0.1	<0.1	<0.1	<0.1	<0.1	<0.1	<0.1	<0.1	<0.1	<0.1	<0.1	<0.1	<0.1
- Chlorothalonil	EPA 3550C 2007 + EPA 8270D 2007	mg/kg	0.1	<0.1	<0.1	<0.1	<0.1	<0.1	<0.1	<0.1	<0.1	<0.1	<0.1	<0.1	<0.1	<0.1	<0.1	<0.1	<0.1	<0.1	<0.1
- Alachlor	EPA 3550C 2007 + EPA 8270D 2007	mg/kg	0.1	<0.1	<0.1	<0.1	<0.1	<0.1	<0.1	<0.1	<0.1	<0.1	<0.1	<0.1	<0.1	<0.1	<0.1	<0.1	<0.1	<0.1	<0.1
- Heptachlor	EPA 3550C 2007 + EPA 8270D 2007	mg/kg	0.1	<0.1	<0.1	<0.1	<0.1	<0.1	<0.1	<0.1	<0.1	<0.1	<0.1	<0.1	<0.1	<0.1	<0.1	<0.1	<0.1	<0.1	<0.1

- Aldrin	EPA 3550C 2007 + EPA 8270D 2007	mg/kg	0.1	<0.1	<0.1	<0.1	<0.1	<0.1	<0.1	<0.1	<0.1	<0.1	<0.1	<0.1	<0.1	<0.1	<0.1	<0.1	<0.1	<0.1	<0.1
- Chlorthal-dimethyl (DCPA)	EPA 3550C 2007 + EPA 8270D 2007	mg/kg	0.1	<0.1	<0.1	<0.1	<0.1	<0.1	<0.1	<0.1	<0.1	<0.1	<0.1	<0.1	<0.1	<0.1	<0.1	<0.1	<0.1	<0.1	<0.1
- Isodrin	EPA 3550C 2007 + EPA 8270D 2007	mg/kg	0.1	<0.1	<0.1	<0.1	<0.1	<0.1	<0.1	<0.1	<0.1	<0.1	<0.1	<0.1	<0.1	<0.1	<0.1	<0.1	<0.1	<0.1	<0.1
- Heptachlor epoxide b	EPA 3550C 2007 + EPA 8270D 2007	mg/kg	0.1	<0.1	<0.1	<0.1	<0.1	<0.1	<0.1	<0.1	<0.1	<0.1	<0.1	<0.1	<0.1	<0.1	<0.1	<0.1	<0.1	<0.1	<0.1
- trans-Chlordane	EPA 3550C 2007 + EPA 8270D 2007	mg/kg	0.1	<0.1	<0.1	<0.1	<0.1	<0.1	<0.1	<0.1	<0.1	<0.1	<0.1	<0.1	<0.1	<0.1	<0.1	<0.1	<0.1	<0.1	<0.1
- o.p'-DDE	EPA 3550C 2007 + EPA 8270D 2007	mg/kg	0.1	<0.1	<0.1	<0.1	<0.1	<0.1	<0.1	<0.1	<0.1	<0.1	<0.1	<0.1	<0.1	<0.1	<0.1	<0.1	<0.1	<0.1	<0.1
- Endosulfan I	EPA 3550C 2007 + EPA 8270D 2007	mg/kg	0.1	<0.1	<0.1	<0.1	<0.1	<0.1	<0.1	<0.1	<0.1	<0.1	<0.1	<0.1	<0.1	<0.1	<0.1	<0.1	<0.1	<0.1	<0.1
- cis-Chlordane	EPA 3550C 2007 + EPA 8270D 2007	mg/kg	0.1	<0.1	<0.1	<0.1	<0.1	<0.1	<0.1	<0.1	<0.1	<0.1	<0.1	<0.1	<0.1	<0.1	<0.1	<0.1	<0.1	<0.1	<0.1
- trans-Nonachlor	EPA 3550C 2007 + EPA 8270D 2007	mg/kg	0.1	<0.1	<0.1	<0.1	<0.1	<0.1	<0.1	<0.1	<0.1	<0.1	<0.1	<0.1	<0.1	<0.1	<0.1	<0.1	<0.1	<0.1	<0.1
- p.p'-DDE	EPA 3550C 2007 + EPA 8270D 2007	mg/kg	0.1	<0.1	<0.1	<0.1	<0.1	<0.1	<0.1	<0.1	<0.1	<0.1	<0.1	<0.1	<0.1	<0.1	<0.1	<0.1	<0.1	<0.1	<0.1
- Dieldrin	EPA 3550C 2007 + EPA 8270D 2007	mg/kg	0.1	<0.1	<0.1	<0.1	<0.1	<0.1	<0.1	<0.1	<0.1	<0.1	<0.1	<0.1	<0.1	<0.1	<0.1	<0.1	<0.1	<0.1	<0.1
- o.p'-DDD	EPA 3550C 2007 + EPA 8270D 2007	mg/kg	0.1	<0.1	<0.1	<0.1	<0.1	<0.1	<0.1	<0.1	<0.1	<0.1	<0.1	<0.1	<0.1	<0.1	<0.1	<0.1	<0.1	<0.1	<0.1

- Endrin	EPA 3550C 2007 + EPA 8270D 2007	mg/kg	0.1	<0.1	<0.1	<0.1	<0.1	<0.1	<0.1	<0.1	<0.1	<0.1	<0.1	<0.1	<0.1	<0.1	<0.1	<0.1	<0.1	<0.1	<0.1
- Endosulfan II	EPA 3550C 2007 + EPA 8270D 2007	mg/kg	0.1	<0.1	<0.1	<0.1	<0.1	<0.1	<0.1	<0.1	<0.1	<0.1	<0.1	<0.1	<0.1	<0.1	<0.1	<0.1	<0.1	<0.1	<0.1
- p.p'-DDD	EPA 3550C 2007 + EPA 8270D 2007	mg/kg	0.1	<0.1	<0.1	<0.1	<0.1	<0.1	<0.1	<0.1	<0.1	<0.1	<0.1	<0.1	<0.1	<0.1	<0.1	<0.1	<0.1	<0.1	<0.1
- o.p'-DDT	EPA 3550C 2007 + EPA 8270D 2007	mg/kg	0.1	<0.1	<0.1	<0.1	<0.1	<0.1	<0.1	<0.1	<0.1	<0.1	<0.1	<0.1	<0.1	<0.1	<0.1	<0.1	<0.1	<0.1	<0.1
- Endrin aldehyde	EPA 3550C 2007 + EPA 8270D 2007	mg/kg	0.1	<0.1	<0.1	<0.1	<0.1	<0.1	<0.1	<0.1	<0.1	<0.1	<0.1	<0.1	<0.1	<0.1	<0.1	<0.1	<0.1	<0.1	<0.1
- Kepone	EPA 3550C 2007 + EPA 8270D 2007	mg/kg	0.1	<0.1	<0.1	<0.1	<0.1	<0.1	<0.1	<0.1	<0.1	<0.1	<0.1	<0.1	<0.1	<0.1	<0.1	<0.1	<0.1	<0.1	<0.1
- Endosulfan sulfate	EPA 3550C 2007 + EPA 8270D 2007	mg/kg	0.1	<0.1	<0.1	<0.1	<0.1	<0.1	<0.1	<0.1	<0.1	<0.1	<0.1	<0.1	<0.1	<0.1	<0.1	<0.1	<0.1	<0.1	<0.1
- p.p'-DDT	EPA 3550C 2007 + EPA 8270D 2007	mg/kg	0.1	<0.1	<0.1	<0.1	<0.1	<0.1	<0.1	<0.1	<0.1	<0.1	<0.1	<0.1	<0.1	<0.1	<0.1	<0.1	<0.1	<0.1	<0.1
- Methoxychlor	EPA 3550C 2007 + EPA 8270D 2007	mg/kg	0.1	<0.1	<0.1	<0.1	<0.1	<0.1	<0.1	<0.1	<0.1	<0.1	<0.1	<0.1	<0.1	<0.1	<0.1	<0.1	<0.1	<0.1	<0.1
- Mirex	EPA 3550C 2007 + EPA 8270D 2007	mg/kg	0.1	<0.1	<0.1	<0.1	<0.1	<0.1	<0.1	<0.1	<0.1	<0.1	<0.1	<0.1	<0.1	<0.1	<0.1	<0.1	<0.1	<0.1	<0.1
- Permethrins-I	EPA 3550C 2007 + EPA 8270D 2007	mg/kg	0.1	<0.1	<0.1	<0.1	<0.1	<0.1	<0.1	<0.1	<0.1	<0.1	<0.1	<0.1	<0.1	<0.1	<0.1	<0.1	<0.1	<0.1	<0.1
- Permethrins-II	EPA 3550C 2007 + EPA 8270D 2007	mg/kg	0.1	<0.1	<0.1	<0.1	<0.1	<0.1	<0.1	<0.1	<0.1	<0.1	<0.1	<0.1	<0.1	<0.1	<0.1	<0.1	<0.1	<0.1	<0.1
Phosphorus pesticides																					

- Diclorvos (DDVP)	EPA 3550C 2007 + EPA 8270D 2007	mg/kg	0.1	<0.1	<0.1	<0.1	<0.1	<0.1	<0.1	<0.1	<0.1	<0.1	<0.1	<0.1	<0.1	<0.1	<0.1	<0.1	<0.1	<0.1	<0.1
- Mevinphos (Phosdrin)	EPA 3550C 2007 + EPA 8270D 2007	mg/kg	0.1	<0.1	<0.1	<0.1	<0.1	<0.1	<0.1	<0.1	<0.1	<0.1	<0.1	<0.1	<0.1	<0.1	<0.1	<0.1	<0.1	<0.1	<0.1
- Demeton-S	EPA 3550C 2007 + EPA 8270D 2007	mg/kg	0.1	<0.1	<0.1	<0.1	<0.1	<0.1	<0.1	<0.1	<0.1	<0.1	<0.1	<0.1	<0.1	<0.1	<0.1	<0.1	<0.1	<0.1	<0.1
- Ethoprophos	EPA 3550C 2007 + EPA 8270D 2007	mg/kg	0.1	<0.1	<0.1	<0.1	<0.1	<0.1	<0.1	<0.1	<0.1	<0.1	<0.1	<0.1	<0.1	<0.1	<0.1	<0.1	<0.1	<0.1	<0.1
- Naled (Dibrom)	EPA 3550C 2007 + EPA 8270D 2007	mg/kg	0.1	<0.1	<0.1	<0.1	<0.1	<0.1	<0.1	<0.1	<0.1	<0.1	<0.1	<0.1	<0.1	<0.1	<0.1	<0.1	<0.1	<0.1	<0.1
- Phorate	EPA 3550C 2007 + EPA 8270D 2007	mg/kg	0.1	<0.1	<0.1	<0.1	<0.1	<0.1	<0.1	<0.1	<0.1	<0.1	<0.1	<0.1	<0.1	<0.1	<0.1	<0.1	<0.1	<0.1	<0.1
- Demeton-O	EPA 3550C 2007 + EPA 8270D 2007	mg/kg	0.1	<0.1	<0.1	<0.1	<0.1	<0.1	<0.1	<0.1	<0.1	<0.1	<0.1	<0.1	<0.1	<0.1	<0.1	<0.1	<0.1	<0.1	<0.1
- Diazinon	EPA 3550C 2007 + EPA 8270D 2007	mg/kg	0.1	<0.1	<0.1	<0.1	<0.1	<0.1	<0.1	<0.1	<0.1	<0.1	<0.1	<0.1	<0.1	<0.1	<0.1	<0.1	<0.1	<0.1	<0.1
- Disulfoton	EPA 3550C 2007 + EPA 8270D 2007	mg/kg	0.1	<0.1	<0.1	<0.1	<0.1	<0.1	<0.1	<0.1	<0.1	<0.1	<0.1	<0.1	<0.1	<0.1	<0.1	<0.1	<0.1	<0.1	<0.1
- methyl-Parathion	EPA 3550C 2007 + EPA 8270D 2007	mg/kg	0.1	<0.1	<0.1	<0.1	<0.1	<0.1	<0.1	<0.1	<0.1	<0.1	<0.1	<0.1	<0.1	<0.1	<0.1	<0.1	<0.1	<0.1	<0.1
- Fenchlorphos (Ronnel)	EPA 3550C 2007 + EPA 8270D 2007	mg/kg	0.1	<0.1	<0.1	<0.1	<0.1	<0.1	<0.1	<0.1	<0.1	<0.1	<0.1	<0.1	<0.1	<0.1	<0.1	<0.1	<0.1	<0.1	<0.1
- Fenthion	EPA 3550C 2007 + EPA 8270D 2007	mg/kg	0.1	<0.1	<0.1	<0.1	<0.1	<0.1	<0.1	<0.1	<0.1	<0.1	<0.1	<0.1	<0.1	<0.1	<0.1	<0.1	<0.1	<0.1	<0.1

- Chlorpyrifos (Dursban)	EPA 3550C 2007 + EPA 8270D 2007	mg/kg	0.1	<0.1	<0.1	<0.1	<0.1	<0.1	<0.1	<0.1	<0.1	<0.1	<0.1	<0.1	<0.1	<0.1	<0.1	<0.1	<0.1	<0.1	<0.1
- Trichloronate	EPA 3550C 2007 + EPA 8270D 2007	mg/kg	0.1	<0.1	<0.1	<0.1	<0.1	<0.1	<0.1	<0.1	<0.1	<0.1	<0.1	<0.1	<0.1	<0.1	<0.1	<0.1	<0.1	<0.1	<0.1
- Merphos	EPA 3550C 2007 + EPA 8270D 2007	mg/kg	0.1	<0.1	<0.1	<0.1	<0.1	<0.1	<0.1	<0.1	<0.1	<0.1	<0.1	<0.1	<0.1	<0.1	<0.1	<0.1	<0.1	<0.1	<0.1
- Stirofos (Tetrachlorvinphos)	EPA 3550C 2007 + EPA 8270D 2007	mg/kg	0.1	<0.1	<0.1	<0.1	<0.1	<0.1	<0.1	<0.1	<0.1	<0.1	<0.1	<0.1	<0.1	<0.1	<0.1	<0.1	<0.1	<0.1	<0.1
- Tokuthion (Prothiofos)	EPA 3550C 2007 + EPA 8270D 2007	mg/kg	0.1	<0.1	<0.1	<0.1	<0.1	<0.1	<0.1	<0.1	<0.1	<0.1	<0.1	<0.1	<0.1	<0.1	<0.1	<0.1	<0.1	<0.1	<0.1
- Bolstar (Sulprofos)	EPA 3550C 2007 + EPA 8270D 2007	mg/kg	0.1	<0.1	<0.1	<0.1	<0.1	<0.1	<0.1	<0.1	<0.1	<0.1	<0.1	<0.1	<0.1	<0.1	<0.1	<0.1	<0.1	<0.1	<0.1
- Azinphos-methyl (Guthion)	EPA 3550C 2007 + EPA 8270D 2007	mg/kg	0.1	<0.1	<0.1	<0.1	<0.1	<0.1	<0.1	<0.1	<0.1	<0.1	<0.1	<0.1	<0.1	<0.1	<0.1	<0.1	<0.1	<0.1	<0.1
- Coumaphos	EPA 3550C 2007 + EPA 8270D 2007	mg/kg	0.1	<0.1	<0.1	<0.1	<0.1	<0.1	<0.1	<0.1	<0.1	<0.1	<0.1	<0.1	<0.1	<0.1	<0.1	<0.1	<0.1	<0.1	<0.1
Formaldehyde	EPA 8315A 1996	mg/kg	<0.50	3.50	<0,50	5.2	109	<0,50	45	480	218	47	13	236	15.2	<0,50	2.48	15.2	45	12.3	1.50
Acetone	EPA 8315A 1996	mg/kg	5	6	200	<5	<5	<5	<5	<5	<5	<5	65	8	14	23	<5	13	<5	42	<5

Table S2. Descriptive analytics of the measurements reported in Table S1. LOD: Limit of Detection.

Determination	Min	Max	Average	Standard Deviation	RDS
pH	3.7	7.8	4.77	15.2	318.8
Dry matter at 105 °C	53.7	95.7	88.1	1550.0	1758.7
Humidity	4.3	46.3	11.9	1550.0	13062.1
Dry matter at 600 °C	0.32	4.0	1.22	17.4	1427.8
Total Cyanides	< LOD	< LOD	-	-	-
Sulfur	< LOD	< LOD	-	-	-
Aluminum	2.5	2540	170.6	5951547	3489506
Antimony	0.78	0.78	0.78	0	0
Silver	< LOD	< LOD	-	-	-
Arsenic	2.3	2.3	2.30	0	0
Barium	0.86	55.6	15.3	3991.1	26098.9
Beryllium	< LOD	< LOD	-	-	-
Boron	1.6	64.7	8.33	3466.1	41620.9
Cadmium	0.34	0.34	0.34	0	0
Calcium	585	54960	4473.1	2.71E+09	60593533
Cobalt	2.7	2.7	2.70	0	0
Total Chromium	0.57	26.6	4.88	552.9	11327.1
Chromium VI	< LOD	< LOD	-	-	-
Iron	6.9	4655	385.6	20412041	5293045
Magnesium	95.2	4275	449.1	15682560	3492040
Manganese	3.2	260	50.4	63591.7	126160
Mercury	< LOD	< LOD	-	-	-
Molybdenium	0.94	0.94	0.94	0	0
Nickel	0.54	13.2	3.83	117.1	3056.2
Lead	0.67	11.5	3.74	115.8	3092.5
Potassium	152	4780	1188.2	19390559	1631973
Copper	0.53	24.9	3.30	525.0	15893.2
Selenium	< LOD	< LOD	-	-	-
Silicon	4.8	70	39.4	7626.4	19348.2
Sodium	8.8	1145	188.0	1561109	830274.1

Tin	0.69	0.69	0.69	0	0
Thallium	< LOD	< LOD	-	-	-
Tellurium	< LOD	< LOD	-	-	-
Titanium	0.67	50.3	7.38	2091.6	28329.4
Vanadium	0.6	8.9	1.24	63.0	5074.3
Zinc	1.3	58.2	9.10	2906.2	31935.8
Total Organic Compound (TOC)	17.8	47.7	43.6	790.2	1812.3
Aliphatic hydrocarbons (C5÷C8)	< LOD	< LOD	-	-	-
Aromatic hydrocarbons (C9÷C10)	< LOD	< LOD	-	-	-
Hydrocarbons (C10÷C40)	325	1770	818.4	2780186	339694.3
Benzene	< LOD	< LOD	-	-	-
Toluene	0.23	1.2	0.68	0.48	70.8
Ethylbenzene	< LOD	< LOD	-	-	-
o-Xylene	< LOD	< LOD	-	-	-
m-Xylene	< LOD	< LOD	-	-	-
p-Xylene	< LOD	< LOD	-	-	-
Styrene	0.57	0.57	0.57	0	0
Isopropylbenzene	< LOD	< LOD	-	-	-
1,2,4-Trimethylbenzene	< LOD	< LOD	-	-	-
1.3.5-Trimethylbenzene	< LOD	< LOD	-	-	-
Chloromethane	< LOD	< LOD	-	-	-
Vinyl Chloride	< LOD	< LOD	-	-	-
1.1-Dichloroethylene	< LOD	< LOD	-	-	-
Dichloromethane	< LOD	< LOD	-	-	-
trans-1.2-Dichloroethylene	< LOD	< LOD	-	-	-
1.1-Dichloroethane	< LOD	< LOD	-	-	-
Trichloromethane (Chloroform)	< LOD	< LOD	-	-	-
cis-1.2-Dichloroethylene	< LOD	< LOD	-	-	-
1.1.1-Trichloroethane	< LOD	< LOD	-	-	-
Carbon tetrachloride	< LOD	< LOD	-	-	-
1.1-Dichloropropene	< LOD	< LOD	-	-	-
Trichloroethylene	< LOD	< LOD	-	-	-
1.2-Dichloroethane	< LOD	< LOD	-	-	-
1.2-Dichloropropane	< LOD	< LOD	-	-	-

cis-1.3-Dichloropropene	< LOD	< LOD	-	-	-
Tetrachloroethylene (PCE)	< LOD	< LOD	-	-	-
1.1.2-Trichloroethane	< LOD	< LOD	-	-	-
trans-1.3-Dichloropropene	< LOD	< LOD	-	-	-
1.1.1.2-Tetrachloroethane	< LOD	< LOD	-	-	-
1.3-Dichloropropane	< LOD	< LOD	-	-	-
1.1.2.2-Tetrachloroethane	< LOD	< LOD	-	-	-
1.2.3-Trichloropropane	< LOD	< LOD	-	-	-
Hexachlorobutadiene	< LOD	< LOD	-	-	-
1.2-Dibromo-3-chloropropane	< LOD	< LOD	-	-	-
Bromochloromethane	< LOD	< LOD	-	-	-
Bromodichloromethane	< LOD	< LOD	-	-	-
Dibromochloromethane	< LOD	< LOD	-	-	-
1.2-Dibromoethane	< LOD	< LOD	-	-	-
Tribromomethane (Bromoform)	< LOD	< LOD	-	-	-
Naphthalene	< LOD	< LOD	-	-	-
Acenaphtilene	< LOD	< LOD	-	-	-
Acenaftene	< LOD	< LOD	-	-	-
Fluorenes	< LOD	< LOD	-	-	-
Fenanthrene	0.23	0.34	0.27	0.008	3.02
Anthracene	< LOD	< LOD	-	-	-
Fluorantene	0.16	0.25	0.20	0.004	2.00
Perylene	0.16	0.16	0.16	0	0
Pyrene	0.12	0.14	0.13	0.0002	0.154
Benzo(a)anthracene	< LOD	< LOD	-	-	-
Crysene	< LOD	< LOD	-	-	-
Benzo(b)fluorantenes	< LOD	< LOD	-	-	-
Benzo(k+j)fluorantenes	< LOD	< LOD	-	-	-
Benzo(e)pyrene	< LOD	< LOD	-	-	-
Benzo(a)pyrene	< LOD	< LOD	-	-	-
Indeno(1.2.3-cd)pyrenees	< LOD	< LOD	-	-	-
Dibenzo(ah)anthracene	< LOD	< LOD	-	-	-
Benzo(ghi)perilene	< LOD	< LOD	-	-	-
Dibenzo(al)pyrene	< LOD	< LOD	-	-	-

Dibenzo(ae)pyrene	< LOD	< LOD	-	-	-
Dibenzo(ai)pyrene	< LOD	< LOD	-	-	-
Dibenzo(ah)pyrene	< LOD	< LOD	-	-	-
Etridiazole	< LOD	< LOD	-	-	-
Chloroneb	< LOD	< LOD	-	-	-
alfa-BHC	< LOD	< LOD	-	-	-
Simazine	< LOD	< LOD	-	-	-
Atrazine	< LOD	< LOD	-	-	-
beta-BHC	< LOD	< LOD	-	-	-
gamma-BHC (Lindane)	< LOD	< LOD	-	-	-
delta-BHC	< LOD	< LOD	-	-	-
Chlorothalonil	< LOD	< LOD	-	-	-
Alachlor	< LOD	< LOD	-	-	-
Heptachlor	< LOD	< LOD	-	-	-
Aldrin	< LOD	< LOD	-	-	-
Chlorthal-dimethyl (DCPA)	< LOD	< LOD	-	-	-
Isodrin	< LOD	< LOD	-	-	-
Heptachlor epoxide b	< LOD	< LOD	-	-	-
trans-Chlordane	< LOD	< LOD	-	-	-
o.p'-DDE	< LOD	< LOD	-	-	-
Endosulfan I	< LOD	< LOD	-	-	-
cis-Chlordane	< LOD	< LOD	-	-	-
trans-Nonachlor	< LOD	< LOD	-	-	-
p.p'-DDE	< LOD	< LOD	-	-	-
Dieldrin	< LOD	< LOD	-	-	-
o.p'-DDD	< LOD	< LOD	-	-	-
Endrin	< LOD	< LOD	-	-	-
Endosulfan II	< LOD	< LOD	-	-	-
p.p'-DDD	< LOD	< LOD	-	-	-
o.p'-DDT	< LOD	< LOD	-	-	-
Endrin aldehyde	< LOD	< LOD	-	-	-
Kepone	< LOD	< LOD	-	-	-
Endosulfan sulfate	< LOD	< LOD	-	-	-
p.p'-DDT	< LOD	< LOD	-	-	-

Methoxychlor	< LOD	< LOD	-	-	-
Mirex	< LOD	< LOD	-	-	-
Permethrins-I	< LOD	< LOD	-	-	-
Permethrins-II	< LOD	< LOD	-	-	-
Diclorvos (DDVP)	< LOD	< LOD	-	-	-
Mevinphos (Phosdrin)	< LOD	< LOD	-	-	-
Demeton-S	< LOD	< LOD	-	-	-
Ethoprophos	< LOD	< LOD	-	-	-
Naled (Dibrom)	< LOD	< LOD	-	-	-
Phorate	< LOD	< LOD	-	-	-
Demeton-O	< LOD	< LOD	-	-	-
Diazinon	< LOD	< LOD	-	-	-
Disulfoton	< LOD	< LOD	-	-	-
methyl-Parathion	< LOD	< LOD	-	-	-
Fenchlorphos (Ronnel)	< LOD	< LOD	-	-	-
Fenthion	< LOD	< LOD	-	-	-
Chlorpyrifos (Dursban)	< LOD	< LOD	-	-	-
Trichloronate	< LOD	< LOD	-	-	-
Merphos	< LOD	< LOD	-	-	-
Stirofos (Tetrachlorvinphos)	< LOD	< LOD	-	-	-
Tokuthion (Prothiofos)	< LOD	< LOD	-	-	-
Bolstar (Sulprofos)	< LOD	< LOD	-	-	-
Azinphos-methyl (Guthion)	< LOD	< LOD	-	-	-
Coumaphos	< LOD	< LOD	-	-	-
Formaldehyde	2.48	480	89.06	241537.1151	271198.4803
Acetone	6	200	46.4	29777.9	64211.1
